# Metabolic and Physiological Regulation of Aspartic Acid-Mediated Enhancement of Heat Stress Tolerance in Perennial Ryegrass

**DOI:** 10.3390/plants11020199

**Published:** 2022-01-13

**Authors:** Shuhan Lei, Stephanie Rossi, Bingru Huang

**Affiliations:** 1 College of Agro-Grassland Science, Nanjing Agricultural University, Nanjing 210095, China; 2017220001@njau.edu.cn; 2 Department of Plant Biology, Rutgers University, New Brunswick, NJ 08901, USA; srossi@scarletmail.rutgers.edu

**Keywords:** perennial ryegrass, heat stress, aspartate, metabolites

## Abstract

Aspartate is the most critical amino acid in the aspartate metabolic pathway, which is associated with multiple metabolic pathways, such as protein synthesis, nucleotide metabolism, TCA cycle, glycolysis, and hormone biosynthesis. Aspartate also plays an important role in plant resistance to abiotic stress, such as cold stress, drought stress, salt stress or heavy metal stress. This study found that the chlorophyll content and antioxidant active enzyme content (SOD, CAT, POD and APX) of perennial ryegrass treated with 2 mM aspartate were significantly higher than those treated with water under heat stress. The electrolyte leakage rate, MDA content and peroxide levels (O^2−^ and H_2_O_2_) of perennial ryegrass treated with aspartate were significantly lower than those of perennial ryegrass treated with water, indicating that exogenous aspartate increases the content of chlorophyll, maintain the integrity of cell membrane system, and enhances SOD-CAT antioxidant pathway to eliminate the oxidative damage caused by ROS in perennial ryegrass under heat stress. Furthermore, exogenous aspartate could enhance the TCA cycle, the metabolism of the amino acids related to the TCA cycle, and pyrimidine metabolism to enhance the heat tolerance of perennial ryegrass.

## 1. Introduction

Heat stress, a main abiotic stress, detrimentally affects the productivity and growth of temperate plants by interrupting metabolic homeostasis and activities of various biochemical processes, such as photosynthesis, respiration, and stress defense [1]. In addition to genetic modification of plants to improve stress tolerance, chemical priming and exogenous application of organic molecules have become well-recognized and promising strategies for mitigating stress damages in various plant species by regulating various metabolic processes [2]. Identification of natural vegetation molecules that can effectively improve heat tolerance will be of great significance for the adaptation of sustainable agricultural and ecological systems to global warming.

Aspartate or aspartic acid (Asp), a basic amino acid for protein generation, serves as a central building block in nitrogen and carbon metabolism for many metabolic processes, such as the biosynthesis of other amino acids, nucleotides, organic acids in the tricarboxylic acid (TCA) cycle, sugars in glycolysis, and hormones, which are all vital for plant growth and stress resistance [3]. Asp plays key roles in the aspartate-glutamate pathway of amino acid metabolism, serving as a common precursor for basic amino acids (e.g., isoleucine, lysine, methionine, threonine), and can be converted into glutamate and asparagine [4]. In addition, Asp assimilates inorganic nitrogen, providing a nitrogen source for synthesizing other nitrogen-containing compounds in plants [5]. Asp can also be converted to organic acids (e.g., oxaloacetate) through the catalytic activities of enzymes, including Asp aminotransferase and prephenate aminotransferase [6,7]. The Asp-derived amino acid pathway is the subject of extensive research regarding plant nutrition, owing to the key roles of Asp in the biosynthesis of other metabolites [8]. Asp is an essential amino acid for cell proliferation and participates in vegetation reactions to abiotic threats [3,9,10,11].

In response to abiotic stress (e.g., cold stress, drought stress, salt stress, and heavy metal stress), the endogenous content of Asp increases or decreases according to plant species and type of stress [9,10,11,12,13]. The accumulation of Asp along with other amino acids, such as proline under salinity stress, is associated with its roles in osmotic adjustment and membrane stabilization [14]. Asp is considered a drought-responsive biomarker due to the rapid changes in its content in plants exposed to stress [3]. Exogenous application of Asp can improve plant tolerance to salinity [13] and cadmium toxicity [15] based on plant growth and physiological responses, although the underlying metabolic mechanisms are unknown. An extensive and most recent literature review regarding how Asp affects plant reactions to diverse stresses (e.g., cold, drought, light, salinity, metal toxicity, nutrient deficiency, diseases) uncovers the essential functions of Asp in modulating plant tolerance against abiotic and biotic stresses [3]. However, Asp-mediated regulation of heat tolerance has been studied. The impacts of Asp on heat endurance and the underlying metabolic pathways controlled by Asp under heat stress are not well-understood. Given the key biofunctions of Asp as a critical metabolic hub to interconnect with diverse metabolic pathways and its reported positive effects on mitigating abiotic stresses in plants, it is hypothesized that exogenous Asp application may enhance heat endurance of cool-season grass species by activating multiple metabolic pathways involved in heat adaptation of plants.

This study was planned to investigate whether Asp may promote heat tolerance in perennial ryegrass, a cool-season grass species extensively utilized as forage and turfgrass, and which metabolic pathways may be regulated by Asp or are most responsive to Asp that can be associated with Asp-mediated enhancement of heat tolerance. Several commonly-used stress tolerance indicators, including leaf chlorophyll content, membrane stability, generation of reactive oxygen species (ROS, H_2_O_2_, superoxide [O^2–^]) and malondialdehyde, and antioxidant enzyme activities, were analyzed to evaluate heat tolerance in Asp-treated plants in contrast with untreated plants under heat stress. Comparative metabolomic analysis of Asp-treated plants and control plants exposed to heat stress was conducted to recognize the Asp-modulated metabolites that may contribute to Asp-mediated enhancement of heat tolerance in perennial ryegrass.

## 2. Results

### 2.1. Physiological Effects of Aspartate on Perennial Ryegrass Tolerance Response to Heat Stress

**Figure 1:**
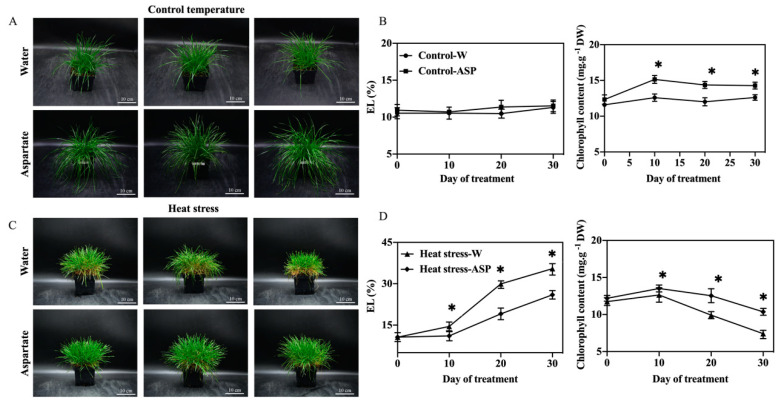
The phenotype of perennial ryegrass applied with water and 2 mM aspartate at 30 d control temperature (**A**) and 30 d heat stress (**C**); effects of exogenous application of aspartate (Asp, 2 mM) on electrolyte leakage (EL) and chlorophyll content in leaves of perennial ryegrass under control temperature (**B**) and heat stress (**D**). White bars in phenotype indicate 10 cm. Vertical bars represent standard errors of a given data point, and * represents significant differences between water and Asp at a given day of control or heat stress treatment based on the LSD test at *p* = 0.05.

Photos depicting the phenotypes of Asp-treated and water-treated (untreated control) plants exposed to 30 d control temperature(non-stress) or 30 d heat stress. The phenotype shows that Asp-treated plants have more green leaves Asp than untreated plants under 30 d heat stress (Figure 1A). There were no significant differences in leaf EL between Asp-treated and untreated plants under non-stress conditions; however, Asp-treated plants had significantly lower EL than untreated plants from 10–30 d of heat stress (Figure 1B). Leaf Chl content was significantly higher in Asp-treated plants compared to untreated control Asp plants at 10 and 30 d of non-stress conditions and at 10 and 30 d of heat stress (Figure 1C).

**Figure 2:**
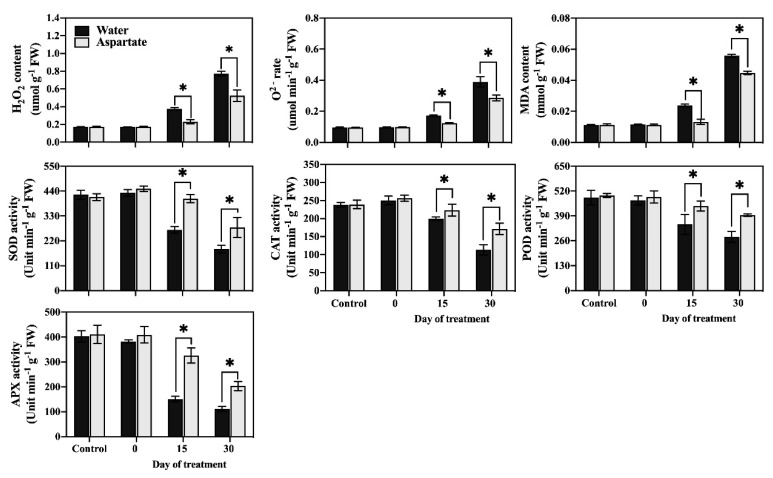
Effects of exogenous application of aspartate (Asp, 2 mM) on hydrogen peroxide (H_2_O_2_) content, superoxide (O^2−)^ production rate, malondialdehyde (MDA) content, and enzymatic activity of superoxide dismutase (SOD), catalase (CAT), peroxidase (POD), and ascorbate peroxidase (APX) in leaves of perennial ryegrass under control temperature and heat stress. Vertical bars over the column represent standard errors of a given data point, and * represents significant differences between water and Asp under control temperature and at a given day of heat stress treatment based on the LSD test at *p* = 0.05.

H_2_O_2_ content (Figure 2), O^2−^ production rate (Figure 2), and MDA content (Figure 2) in leaves did not differ between Asp-treated and untreated plants under non-stress control conditions. At 15 d and 30 d of heat stress, Asp-treated plants maintained a significantly lower production rate of O^2−^, H_2_O_2_ content, and MDA content.

**Figure 3:**
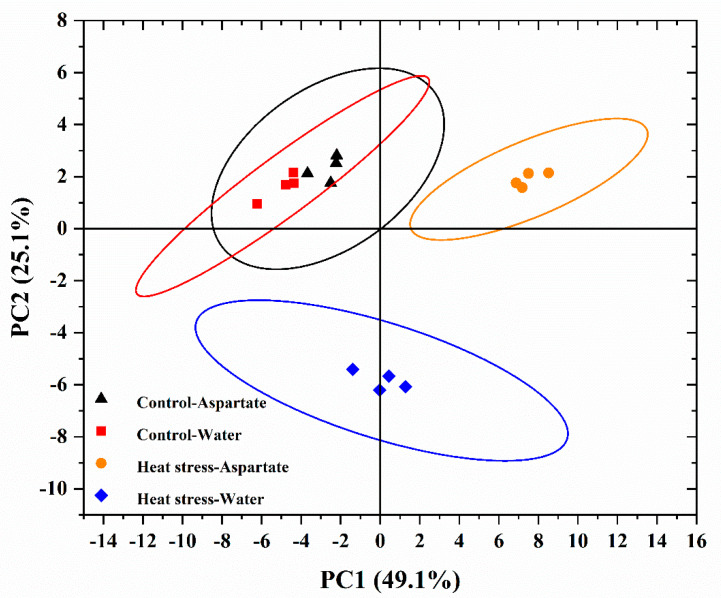
Principal component analysis (PCA) of differentially regulated metabolites by water and aspartate (Asp, 2 mM) treatments of perennial ryegrass exposed to control temperature and heat stress conditions.

The activity of four antioxidant enzymes (CAT, POD, APX, and SOD) remained unchanged in response to Asp application under non-stress control conditions (Figure 3). The activities of CAT, POD, APX, and SOD in Asp-treated plants were significantly higher than those of untreated control plants at 15 d and 30 d of heat stress.

Analysis of commonly used physiological and biochemical parameters (EL, Chl, ROS content, MDA content, and antioxidant enzyme activity) for the evaluation of heat stress tolerance demonstrated that Asp application improved the heat tolerance of perennial ryegrass.

### 2.2. Metabolites Differentially Responsive to Aspartate Application in Perennial Ryegrass

To find out which key metabolites were regulated by Asp in association with Asp-mediated enhancement of heat tolerance in perennial ryegrass, all metabolites having differential changes in their endogenous content in response to Asp application relative to the untreated control under non-stress control temperature or heat stress (30 d) were analyzed. There were 52 metabolites responsive to heat and Asp (Table 1). Principal component analysis distinguished the groups of metabolites that were specifically responsive to Asp under non-stress control temperature or heat stress (25.1%, PC2) and those that were affected by heat stress for either Asp-treated plants or untreated control plants (49.1%, PC1) (Figure 3).

**Figure 4:**
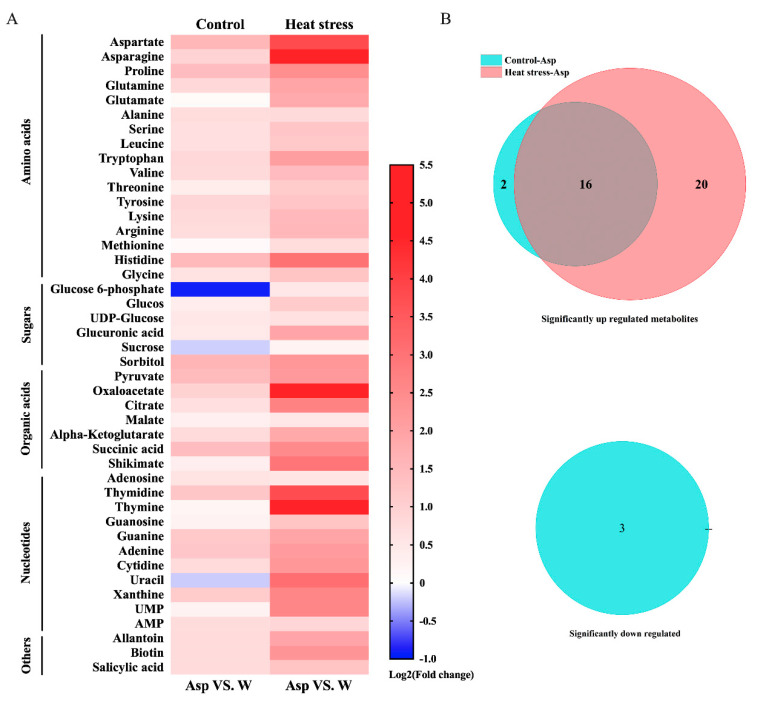
Heat map showing fold-changes for metabolites responsive to aspartate (Asp, 2 mM) with increases (up-regulation showing in red bars) or decreases (down-regulation showing in blue bars) in metabolite content in plants due to Asp treatment in comparison to metabolite content in the water-treated plants of perennial ryegrass under control temperature and heat stress (**A**). Venn diagram analysis demonstrating the number of significant up-regulated and down-regulated metabolites by Asp in perennial ryegrass under control temperature and heat stress (**B**).

A total of 44 metabolites were found to be responsive to Asp in perennial ryegrass under non-stress control temperature and heat stress, including sugars, organic acids, amino acids, and nucleotides (Supplementary Material Table S1). The heat map depicts metabolites with increased content (fold change > 1 indicated up-regulation shown by red bars) or decreased content (fold change < 1 indicated down-regulation shown by blue bars) in Asp-treated plants in comparison to the untreated control plants under non-stress control temperature or heat stress (Figure 4). Most metabolites exhibited up-regulation, while three metabolites were down-regulated by Asp under non-stress control conditions. Under heat stress, all metabolites were up-regulated by Asp (Figure 4A). The Venn diagram demonstrates that among 41 metabolites with significant increases in their content due to Asp treatment, 20 metabolites (6 carbohydrates, 10 amino acids, 4 nucleotides) were significantly increased or regulated only by Asp treatment under heat stress (Figure 4B).

### 2.3. The Key Differential Metabolites and Metabolic Pathways Regulated by Aspartate in Perennial Ryegrass Exposed to Control Temperature and Heat Stress

**Figure 5:**
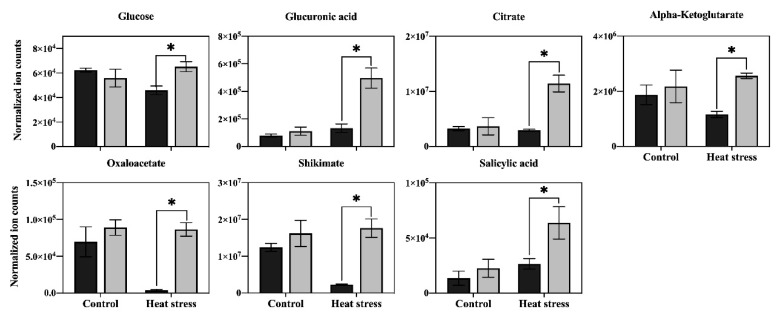
Effects of exogenous application of aspartate (Asp, 2 mM) on the content of sugars and organic acids in leaves of perennial ryegrass under control temperature and heat stress (30 d of treatment). Vertical bars over the column represent standard errors of a given data point, and * represents significant differences between water and Asp under control temperature and heat stress based on the LSD test at *p* = 0.05.

**Figure 6:**
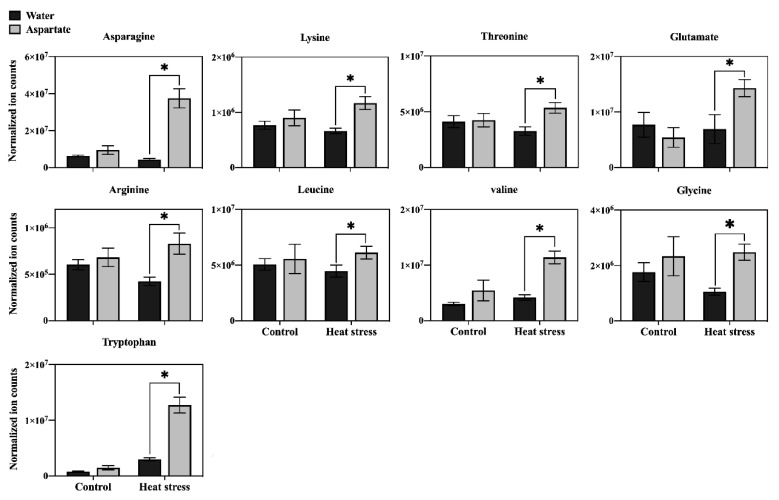
Effects of exogenous application of aspartate (Asp, 2 mM) on the content of amino acids in leaves of perennial ryegrass under control temperature and heat stress (30 d of treatment). Vertical bars over the column represent standard errors of a given data point, and * represents significant differences between water and Asp under control temperature and heat stress based on the LSD test at *p* = 0.05.

**Figure 7:**
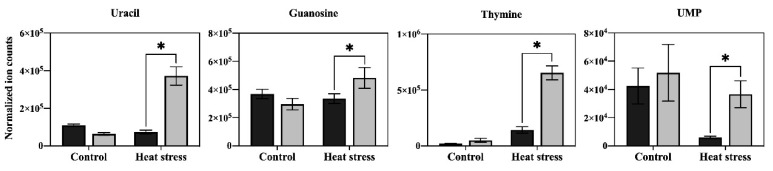
Effects of exogenous application of aspartate (Asp, 2 mM) on the content of nucleotides in leaves of perennial ryegrass under control temperature and heat stress (30 d of treatment). Vertical bars over the column represent standard errors of a given data point, and * represents significant differences between water and Asp under control temperature and heat stress based on the LSD test at *p* = 0.05.

In order to determine the major metabolic pathways regulated by Asp that may improve heat tolerance in perennial ryegrass, the 20 metabolites specifically responsive to Asp under heat stress were further analyzed. The content of glucose and six organic acids (oxaloacetate, glucuronic acid, citrate, ketoglutarate, shikimate, and salicylic acid) was significantly higher in Asp-treated plants than untreated controls under heat stress, while the content of those metabolites did not show significant changes in response to Asp treatment under non-stress conditions (Figure 5). The content of nine amino acids (asparagine, lysine, threonine, glutamate, arginine, leucine, valine, glycine, and tryptophan) increased significantly in response to Asp treatment under heat stress, but none of those amino acids showed significant changes to Asp under non-stress conditions (Figure 6). The content of four nucleotides (uracil, guanosine, thymine, and uridine monophosphate [UMP]) was significantly higher in Asp-treated plants relative to the untreated controls under heat stress, but not under non-stress conditions (Figure 7).

**Figure 8:**
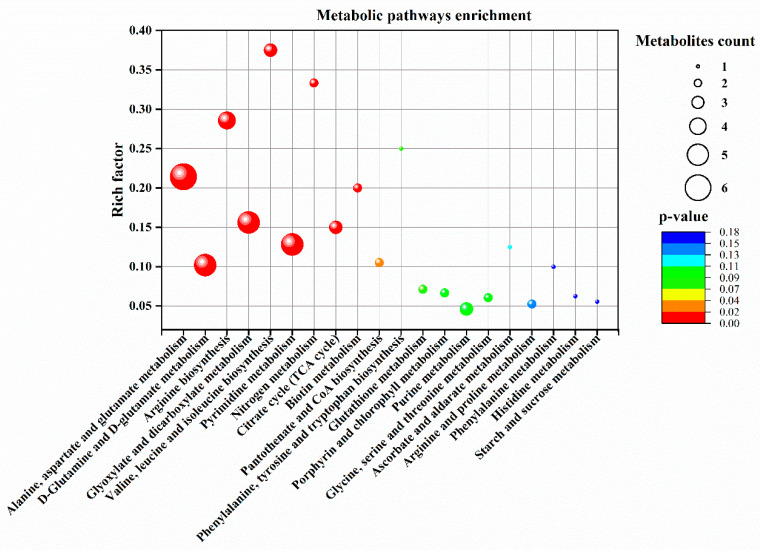
Metabolic pathways enrichment analysis of 20 significantly up-regulated metabolites by aspartate (Asp, 2 mM) in perennial ryegrass leaves at 30 d of heat stress. The bubble size represents the number of Asp-responsive metabolites enriched in a given metabolic pathway. The color of bubbles represents the level of significance based on the LSD test (*p* = 0.05).

**Figure 9:**
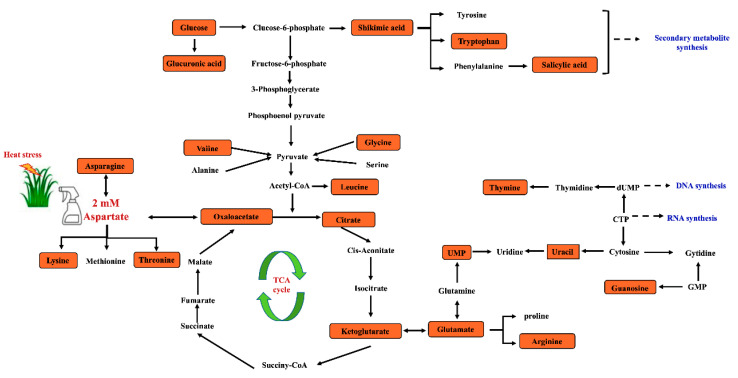
Schematic diagram illustrating metabolic pathways and specific metabolites regulated by aspartate in perennial ryegrass exposed to heat stress in relation to Asp-enhanced heat tolerance. The orange boxes represent significantly up-regulated metabolites by aspartate treatment.

Pathway enrichment analysis was performed for metabolites that exhibited significant up-regulation by Asp in plants exposed to 30 d of heat stress (Figure 8). The Asp metabolites up-regulated by Asp under heat stress were enriched in 20 metabolic processes, with a majority of metabolites enriched in the following seven pathways: (1) alanine, aspartate, and glutamate metabolism; (2) glutamine and glutamate metabolism; (3) arginine biosynthesis; (4) glycolate and dicarboxylate metabolism; (5) valine, leucine, and isoleucine biosynthesis; (6) pyrimidine metabolism, and (7) citric acid cycle (TCA cycle of respiration). Details of the metabolites up-regulated by Asp and associated metabolic pathways in perennial ryegrass exposed to heat stress are demonstrated in Figure 9.

## 3. Discussion

Loss of chlorophyll and membrane stability and induction of ROS production causing lipid peroxidation are typical symptoms of heat stress damage in plants [1]. In this study, exogenous application of Asp allowed plants to maintain lower EL, ROS (O_2_^−^ and H_2_O_2_) production, MDA content, and higher Chl content and antioxidant enzyme (SOD, CAT, POD, and APX) activities. Asp-mediated suppression of cadmium-induced oxidative stress has been associated with the positive effects of Asp on the activities of antioxidant enzymes (SOD, POD, CAT, and APX) in rice (*Oryza sativa*) [15]. Results of the current study suggested that Asp effectively alleviated heat damages and improved heat tolerance in perennial ryegrass by suppressing the loss of Chl, promoting membrane stability, and activating enzymatic antioxidants to reduce oxidative damages induced by heat stress.

Heat stress disrupts various metabolic processes, leading to the death of plants [1]. Comparative metabolic analysis of Asp-treated and untreated control plants revealed that application of Asp helped perennial ryegrass plants maintain metabolic activity under heat stress, as manifested by the increased content or up-regulation of 20 metabolites only under heat stress, including glucose, six organic acids (oxaloacetate, glucuronic acid, citrate, ketoglutarate, shikimate, and salicylic acid), nine amino acids (asparagine, lysine, threonine, glutamate, arginine, leucine, valine, glycine, and tryptophan), and four nucleotides (uracil, guanosine, thymine, and UMP). The metabolites uniquely up-regulated by Asp were mainly enriched in seven metabolic pathways, including (1) alanine, aspartate, and glutamate metabolism, (2) glutamine and glutamate metabolism, (3) arginine biosynthesis, (4) glycolate and dicarboxylate metabolism, (5) valine, leucine and isoleucine biosynthesis, (6) pyrimidine metabolism, and (7) citric acid cycle (TCA cycle of respiration), which could be the prominent metabolic processes involved in Asp-mediated enhancement of heat tolerance in perennial ryegrass. The major metabolites and associated metabolic pathways regulated by Asp that may confer Asp-mediated heat tolerance enhancement are discussed below.

Glucose is a product of photosynthesis that is involved in various metabolic processes, particularly in energy production and the synthesis of organic acids in glycolysis and the TCA cycle during cellular respiration, where it is further metabolized into other products, such as amino acids [16]. The increase of glucose content facilitated by exogenous application of Asp has been relevant to the improvement in plant tolerance to abiotic stresses because it increases the content of antioxidants and osmotic adjustment to enhance stress protection [16]. The accumulation of glucose in the leaves of perennial ryegrass exogenously treated with Asp could contribute to Asp-mediated enhancement of heat tolerance by regulating sugar-related energy metabolism and stress protection (Figure 9).

Glutamate acid is an important component of several pathways and links amino acid and respiration metabolism together by serving as a precursor of proline and arginine and converting to and from α-ketoglutarate in the TCA cycle [17]. The accumulation of glutamate in leaves of creeping bentgrass (*Agrostis stoloniferaand*) tall fescue (*Festuca arundinacea*) has been positively correlated with heat and drought tolerance [18,19]. Arginine is an important precursor of nitric oxide and compounds involved in the synthesis of polyamines, participates in various physiological and biochemical processes of plants, and significantly contributes to plant growth, development, and stress tolerance [20]. In this study, exogenous application of Asp resulted in increased accumulation of glutamate and arginine under heat stress, suggesting the activation of glutamate metabolism and the TCA cycle of respiration could have contributed to Asp-mediated enhancement of heat tolerance in perennial ryegrass.

Leucine is a precursor of acetyl-CoA, while valine is a precursor of succinyl-CoA, and both are involved in the TCA cycle of respiratory metabolism. Increased leucine and valine content in tomato (*Lycopersicon esculentum*) leaves exogenously treated with Asp has been positively correlated with salt tolerance [21]. In the current study, the content of valine and leucine increased in leaves of Asp-treated perennial ryegrass under heat stress (Figure 9). This indicates that exogenous application of aspartate may have promoted activity of the TCA cycle for the production of respiratory metabolites and energy, supporting plant growth under heat stress.

Content of three key organic acids in the TCA cycle (citrate, ketoglutarate, and oxaloacetate) was also elevated in leaves of perennial ryegrass treated with Asp under heat stress (Figure 9), further suggesting the activation of the TCA cycle for respiratory metabolism. Exogenous application of citrate enhanced tall fescue’s heat resistance and antioxidant capacity [22]. An accumulation of oxaloacetate in the roots of eggplant (*Solanum melongena*) [23] was associated with tolerance to iron stress [24]. The content of α-ketoglutarate significantly increased relative to cadmium tolerance in bermudagrass (*Cynodon dactylon*). Results of the current study indicate that exogenous application of Asp could promote the accumulation of citrate, α-ketoglutarate, and oxaloacetate, improving the function of the TCA cycle and influencing leaf respiration under heat stress for enhancement of heat tolerance in perennial ryegrass.

Several nucleotides (uracil, UMP, guanosine, and thymine) involved in pyrimidine metabolism also increased in response to Asp application in perennial ryegrass under heat stress (Figure 9). Uracil and UMP are associated with RNA synthesis [25]. The content of uracil has been shown to significantly decrease in soybean (*Glycine max*) subjected to heat or drought stress [26]. Mutants of UMP kinase became more sensitive to cold stress in rice (*O. sativa*), as manifested by a reduction in Chl content and photosynthesis [27]. Thymine and guanosine are important components of DNA, and it has been exhibited that thymine has a positive effect on drought tolerance of wheat (*Triticum aestivum*), as drought-tolerant cultivars had a higher level of thymine than that of drought-sensitive cultivars [28,29]. The increased content of uracil, UMP, guanosine, and thymine by Asp could enhance pyrimidine metabolism in perennial ryegrass under heat stress, which may contribute to the maintenance of RNA and DNA synthesis, supporting plant growth and defense against heat stress.

In summary, this study demonstrated that exogenous application of Asp effectively enhanced heat tolerance in perennial ryegrass, as manifested by increased Chl content, cell membrane stability, and activation of the SOD and CAT antioxidant pathways for eliminating oxidative damage caused by ROS under heat stress. Asp-mediated enhancement of heat tolerance could involve the up-regulation or activation of amino acid and organic acid metabolism in the TCA pathway of respiration, as well as pyrimidine metabolism. Further studies may investigate whether Asp could serve as a signaling molecule or biomarker for identifying candidate genes associated with Asp-mediated regulation of those metabolic pathways for improving plant tolerance to heat stress and other abiotic stresses.

## 4. Materials and Methods

### 4.1. Plant Materials and Growth

Perennial ryegrass (cv. ‘Pinnacle’) seedlings were grown in 10-cm-long plastic pots (diameter 20 cm) stuffed with 1:1 sand-soil (*v*/*v*). The seedlings were cultivated in the greenhouse at Rutgers University for 60 d at 24/19 °C (day/night) under natural solar irradiation and supplemental sodium lights of ~760 µmol m^−2^·s^−1^ photosynthetically active radiation (PAR) at canopy level. During this 60-d period, the plants were fertilized slightly with 1/2 Hoagland’s solution [30] and irrigated every two days. The plants were clipped once per week and kept at a canopy height of 8–9 cm. After the 60 d, the plants were removed to artificial climate chambers (Chagrin Falls, OH, USA) at 25/20 °C (day/night) and 14-h PAR each day at 650 µmol m^−2^·s^−1^.

### 4.2. Experimental Design and Treatments

A 2 mM Asp solution or water (control) was adopted as a foliar spray to the plants exposed to two temperature treatments (non-stress and heat stress) on the day prior to the treatment and then every 7 d during stress treatment. The control and heat treatment temperatures were 25/20 and 35/30 °C (day/night), respectively. The Asp concentration of 2 mM was selected as the optimally effective rate based on a preliminary screening experiment in which 0.1, 1, 2, 5, 10, and 15 mM Asp were foliar-applied weekly to perennial ryegrass, which was exposed to 20 d of heat stress at 35/30 °C (day/night). In the 20 d of heat stress, plants treated with 2 mM Asp produced significantly more tillers than the untreated control (9 vs. 6 tillers per plant).

Each temperature treatment was repeated four times in four growing chambers controlled in a 14-h photoperiod under 650 µmol·m^−2^·s^−1^ PAR. The untreated control (water only) and Asp treatment were each planted in eight replicate pots, which were randomly put into either the control or treated chamber. Watering was ensured twice a day to maintain soil capacity until draining from the pot bottom, and fertilization once a week was applied with 1/2 Hoagland’s solution [30].

### 4.3. Physiological Evaluation of Heat Tolerance

Cell membrane stability was evaluated by leaf electrolyte leakage (EL). In brief, fresh leaves (0.2 g) were put into each test tube filled with 20 mL of deionized water and then vibrated for 24 h at ambient temperature, followed by detection of the initial aqueous conductivity (Cinitial) with a conductivity analyzer (YSI Inc., Yellow Springs, OH, USA). The leaves were then boiled at 121 °C for 20 min and shaken for another 24 h to measure maximum conductivity (Cmax). Percent EL was computed as Cinitial/Cmax ×100 [31].

For leaf chlorophyll (Chl) detection, fresh leaves (0.1 g) were immersed light-free in dimethyl sulfoxide (DMSO, 10 mL) for 72 h. The absorbance of the Chl extract at 663 and 645 nm was determined by a spectrophotometer (Thermo Fisher Scientific, Madison, WI, USA). Chl content was computed by a reported formula [32].

### 4.4. Quantification of ROS

The O^2−^ and H_2_O_2_ production rates were detected using reported methods [33,34]. As for O^2−^, fresh leaves (0.1 g) were ground into powder in liquid N2 and homogenized in potassium phosphate buffer (PBS, 3 mL, 65 mM, pH 7.8), followed by 15 min of centrifugation at 10,000× *g* and 4 °C. Then the supernatant was cultured first in a solution containing PBS (pH 7.8) and 10 mM hydroxylamine hydrochloride at 25 °C for 20 min, and then in 1 mL of 58 mM sulfonamide and 1 mL of 7 mM naphthylamine for 20 min. The resulting solution was added into 3 mL of chloroform for 3 min of centrifugation at 10,000× *g*. Then, the absorbance at 530 nm of the upper phase was monitored by the spectrophotometer. The O^2−^ production rate was calculated according to Elstner (1976) [33].

As for H_2_O_2_ content, fresh leaves (0.5 g) were ground into powder in liquid N_2_, homogenized in 5 mL of 0.1% (*w*/*v*) trichloroacetic acid (TCA), and then centrifuged at 12,000× *g* for 15 min. The supernatant (0.5 mL) was mixed with 0.5 mL of 10 mM PBS (pH 7.0) and 1 mL of 1 M KI, followed by 15 min of dark cultivation at 28 °C. The absorbance at 390 nm of the incubated solution was detected by the spectrophotometer. H_2_O_2_ content was determined from the standard curve plotted with known H_2_O_2_ levels [34].

### 4.5. Analysis of Antioxidant Enzyme Activities and Lipid Peroxidation Product Content

Leaf tissues (0.3 g) were ground into powder in liquid N_2_. Then a crude enzyme solution was extracted from the powder and homogenized in 3 mL PBS (50 mM, pH 7.8, 1% polyvinylpyrrolidone and 0.2 mM ethylenediaminetetraacetic acid), followed by 20 min of centrifugation at 15,000× *g* and 4 °C. Then the activities of catalase (CAT), peroxidase (POD), ascorbate peroxidase (APX), and superoxide dismutase (SOD) in the supernatant were monitored according to some existing methods [35]. The absorbance of the extracts at 560, 470, 240, and 290 nm for SOD, POD, CAT, and APX, respectively, was measured by the spectrophotometer.

Malondialdehyde (MDA) resulting from membrane lipid peroxidation was monitored as described by Heath (1968) [36]. Fresh leaf (0.5 g) powder ground in liquid N_2_ was added with 6 mL of 5% TCA and centrifuged at 10,000× *g* and 4 °C for 20 min. The supernatant (1 mL) was blended in a solution containing 2 mL of 20% TCA and 0.5% thiobarbituric acid. After incubation at 95 °C for 30 min, the resulting solution was centrifuged at 10,000× *g* for 10 min. The supernatant’s absorbance at 532 and 600 nm was measured with the spectrophotometer. MDA content was determined using an extinction factor of 155 mM^−1^·cm^−1^ according to Heath and Packer (1968) [36].

### 4.6. Metabolic Analysis

#### 4.6.1. Metabolite Extraction

Leaves were sampled from plants after 30 d of heat stress treatment and lyophilized in a FreeZone 4.5 device (Labconco, Kansas City, MO, USA), reaching constant weights. The samples were then ground to fine powder, which (20 mg) was resuspended in 1 mL of a 40:40:20 (*v*:*v*:*v*) methanol:acetonitrile:water solution with 0.1% formic acid. Then after incubation at room temperature for 10 min, the solutions were neutralized by adding 50 ul of a 15% (m/v) NH_4_HCO_3_ solution. The samples were further diluted 4× in 40:40:20 methanol:acetonitrile:water and centrifuged at 4 °C and 16,000× *g* for 10 min. The supernatant was transferred to a clean tube, added with internal standards, and stored at -80 °C until further analysis. The internal standards are a mixture of stable isotope-labeled compounds, including ^2^H_8_-Lysine, ^2^H_3_-Malate, ^13^C_3_,^15^N-Serine, ^2^H_5_,^15^N_2_-Glutamine, ^13^C_2_,^15^N-Glycine, ^2^H_4_-Thymine, ^15^N_4_-Inosine, ^2^H_8_-Methionine, ^13^C_6_-Adipic acid, ^2^H_6_-Ornithine, ^2^H_6_-Myo-inositol, ^2^H_4_,^15^N-Alanine, ^13^C_6_, ^2^H_7_-Glucose, and ^2^H_9_-Phosphocholine.

#### 4.6.2. LC/MS Analysis

HILIC separation was performed on a Vanquish Horizon UHPLC system (Thermo Fisher Scientific) with an XBridge BEH Amide column (150 × 2.1 mm2, 2.5 μm particle size, Waters, Milford, MA, USA) using a gradient of solvent A and solvent B (consisting of 95%:5% and 20%:80%, respectively, H_2_O: acetonitrile with 20 mM acetic acid, 40 mM NH_4_OH, pH 9.4). The gradient (for solvent B) was 100% (0, 3 min), 90% (3.2, 6.2 min), 80% (6.5, 10.5 min), 70% (10.7, 13.5 min), 45% (13.7, 16 min), and 100% (16.5, 22 min). The flow rate was 300 μL/min. The column and autosampler were controlled at 25 and 4 °C, respectively. The injection volume was 5 μL. MS was scanned in both cation and anion modes with a resolution of 70,000 at *m/z* 200, gain-auto-control of 3 × 106 and *m/z* scan from 72 to 1000. Metabolite data were acquired using the MAVEN package (PMID 21049934, mass accuracy window: 5 ppm). Metabolite annotation was based on the accurate mass and retention time matched to our in-house metabolite library.

### 4.7. Statistical Analysis

A two-way analysis of variance was run on SPSS 13.0 (SPSS Inc., Chicago, IL, USA) to test the significance between main treatment effects and the interaction of plant lines and heat stress. Fisher’s protected least significant difference (LSD) was used to test significance at p = 0.05 on a specific day of stress treatment. A heat map was drawn on GraphPad prism 8. Principal component analysis (PCA), Venn diagram, and metabolic pathway enrichment analysis diagram were finished on Origin 2019 (OriginLab Corporation, Northampton, MA, USA). PCA analysis was performed by Origin (PCA Analysis app) and data preparation and analysis steps were performed according to the Origin website requirements (https://www.originlab.com/fileExchange/details.aspx?fid=328, accessed on 1 July 2021). Metabolic pathway enrichment analysis used MetaboAnalyst 5.0 website (https://www.metaboanalyst.ca/MetaboAnalyst/ModuleView.xhtml, accessed on 1 September 2021).

## Figures and Tables

**Table 1:** Metabolites in plant leaf tissues for water treatment and 2 mM aspartate treatment of perennial ryegrass exposed to control temperature and heat stress conditions.

No.	RT (min)	Metabolites	*m/z*	No.	RT (min)	Metabolites	*m/z*
1	2.66	Adenine	134.05	27	3.88	Leucine	130.09
2	2.64	Adenosine	266.09	28	12.63	Lysine	145.10
3	3.04	Allantoin	157.04	29	8.79	Malate	133.01
4	6.48	Alanine	88.04	30	4.45	Methionine	148.04
5	7.34	Alpha-Ketoglutarate	145.01	31	8.54	Oxaloacetate	131.00
6	9.40	AMP	346.06	32	2.94	Phenylalanine	164.07
7	8.70	Aspartate	132.03	33	5.85	Proline	114.06
8	7.54	Asparagine	131.05	34	2.54	Pyruvate	87.01
9	12.43	Arginine	173.10	35	5.92	Ribitol	151.06
10	5.16	Biotin	243.08	36	1.36	Salicylic acid	137.02
11	11.51	Citrate	191.02	37	7.49	Serine	104.04
12	3.90	Cytidine	242.08	38	7.05	Shikimate	173.05
13	8.88	Citrulline	174.09	39	5.73	Sorbitol	181.07
14	7.55	Fumarate	115.00	40	8.28	Succinic acid	117.02
15	5.77	Glucose	179.06	41	7.14	Sucrose	341.11
16	10.58	Glucose 6-phosphate	259.02	42	7.08	Thiamine	263.10
17	11.12	Glucose 1-phosphate	259.09	43	6.76	Threonine	118.05
18	8.23	Glucuronic acid	193.04	44	2.06	Thymidine	241.08
19	8.48	Glutamate	146.05	45	1.99	Thymine	125.04
20	7.30	Glutamine	145.06	46	4.39	Tryptophan	203.08
21	4.17	Guanosine	282.08	47	4.94	Tyrosine	180.07
22	3.38	Guanine	150.04	48	9.60	UDP-Glucose	565.05
23	7.01	Glycine	74.02	49	9.46	UMP	323.03
24	9.78	Histidine	154.06	50	2.12	Uracil	111.02
25	4.33	Isoleucine	130.89	51	5.38	Valine	115.00
26	3.87	Lactate	89.02	52	3.92	Xanthine	151.03

RT, retention time; *m/z*, mass to charge ratio.

## Data Availability

No new data were created or analyzed in this study. Data sharing is not applicable to this article.

## Supplementary Materials

The following supporting information can be downloaded at: https://www.mdpi.com/article/10.3390/plants11020199/s1, Table S1: Normalized ion counts data of 44 metabolites responsive to Asp in perennial ryegrass under non-stress control temperature and heat stress conditions.
